# Celastrol ameliorates hypoxic-ischemic brain injury in neonatal rats by reducing oxidative stress and inflammation

**DOI:** 10.1038/s41390-024-03246-9

**Published:** 2024-05-20

**Authors:** Yingying Hu, Yan Nan, Hongzhou Lin, Qianlei Zhao, Tingting Chen, Xiaoyue Tao, Bingqing Ding, Liying Lu, Shangqin Chen, Jianghu Zhu, Xiaoling Guo, Zhenlang Lin

**Affiliations:** 1 Department of Neonatology, The Second Affiliated Hospital and Yuying Children’s Hospital of Wenzhou Medical University, Wenzhou, Zhejiang China; 2 Key Laboratory of Structural Malformations in Children of Zhejiang Province, The Second Affiliated Hospital and Yuying Children’s Hospital of Wenzhou Medical University, Wenzhou, Zhejiang China; 3 Key Laboratory of Perinatal Medicine of Wenzhou, The Second Affiliated Hospital and Yuying Children’s Hospital of Wenzhou Medical University, Wenzhou, Zhejiang China; 4 Department of Pediatrics, The Second Affiliated Hospital and Yuying Children’s Hospital of Wenzhou Medical University, Wenzhou, Zhejiang China; 5 Basic Medical Research Center, The Second Affiliated Hospital and Yuying Children’s Hospital of Wenzhou Medical University, Wenzhou, Zhejiang China; 6 Key Laboratory of Children Genitourinary Diseases of Wenzhou, The Second Affiliated Hospital and Yuying Children’s Hospital of Wenzhou Medical University, Wenzhou, Zhejiang China

## Abstract

**Background:**

Hypoxic-ischemic encephalopathy (HIE) is caused by perinatal hypoxia and subsequent reductions in cerebral blood flow and is one of the leading causes of severe disability or death in newborns. Despite its prevalence, we currently lack an effective drug therapy to combat HIE. Celastrol (Cel) is a pentacyclic triterpene extracted from *Tripterygium Wilfordi* that can protect against oxidative stress, inflammation, and cancer. However, whether Cel can alleviate neonatal hypoxic-ischemic (HI) brain damage remains unclear.

**Methods:**

Here, we established both in vitro and in vivo models of HI brain damage using CoCl_2_-treated PC12 cells and neonatal rats, respectively, and explored the neuroprotective effects of Cel in these models.

**Results:**

Analyses revealed that Cel administration reduced brain infarction size, microglia activation, levels of inflammation factors, and levels of oxidative stress markers by upregulating levels of p-AMPKα, Nrf2, HO-1, and by downregulating levels of TXNIP and NLRP3. Conversely, these beneficial effects of Cel on HI brain damage were largely inhibited by AMPKα inhibitor Compound C and its siRNA.

**Conclusions:**

We present compelling evidence that Cel decreases inflammation and oxidative stress through the AMPKα/Nrf2/TXNIP signaling pathway, thereby alleviating neonatal HI brain injury. Cel therefore represents a promising therapeutic agent for treating HIE.

**Impact:**

- We firstly report that celastrol can ameliorate neonatal hypoxic-ischemic brain injury both in in vivo and in vitro, which represents a promising therapeutic agent for treating related brain injuries.
- Celastrol activates the AMPKα/Nrf2/TXNIP signaling pathway to relieve oxidative stress and inflammation and thereby alleviates neonatal hypoxic-ischemic brain injury.

## Introduction

Hypoxic-ischemic encephalopathy (HIE) is a type of brain damage caused by acute asphyxia of the brain during the perinatal period. The reported incidence of HIE is approximately 1.5/1000 live births in developed countries and 10–20/1000 live births in underdeveloped countries.^1–3^ HIE is associated with high levels of mortality and a variety of life-long morbidities.^4–6^ Currently, hypothermia therapy is the only clinical standard therapy for neonatal HIE. However, this therapeutic method has limited success in reducing the incidence of death and neurological dysfunction due to a short therapeutic window. Moreover, a recent study found a high prevalence of HIE with poor prognoses, affecting about 20–30% of neonates who underwent therapeutic hypothermia.^7^ Thus, it is critical to develop more effective treatment strategies to improve the prognosis of those impacted by neonatal hypoxic-ischemic (HI) brain damage.

The pathophysiology of neonatal HIE is driven primarily by oxidative stress, mitochondrial dysfunction, glutaminergic excitotoxicity, inflammation, and apoptosis.^8–12^ Oxidative stress plays a particularly important role in the pathogenesis and pathophysiological changes associated with HI brain injury. Both oxidative stress and reactive oxygen species (ROS) produced by HI can damage neurons, leading to lipid peroxidation, protein oxidation, and DNA damage.^13^ Interrupted metabolism results in decreased levels of antioxidant enzymes, which then contribute to the accumulation of ROS.^14^ Inflammation is also a major risk factor for HI brain damage. Inflammatory responses to HI injury are characterized by peripheral leukocyte invasion, microglia, astrocyte activation, and the release of proinflammatory mediators.^5^ Therefore, therapeutic approaches that target oxidative stress and inflammation may be efficient strategies for ameliorating impairments resulting from HI brain injury.

Celastrol (Cel) is a pentacyclic triterpene extracted from *Tripterygium Wilfordi* (thunder god vine). Since 2015, it has attracted increasing research attention due to its beneficial effects on various diseases such as reducing weight in obese patients.^15^ So far, intensive studies have found that celastrol is also a potent neuroprotective agent with anti-oxidative, anti-inflammatory, and anti-apoptotic effects.^15–17^ Many studies have shown that Cel protects against neurodegenerative diseases by reducing the production of neuroinflammatory factors, inhibiting the activation of microglia,^18^ and suppressing nitric oxide (NO) generation.^19,20^ Besides, Cel could minimize damage caused by transient global cerebral ischemia (or permanent cerebral ischemia) by promoting microglia/macrophage M2 polarization^18^ and regulating lipid metabolism^21^ via NF-κB and HMGB1 pathways to exert anti-inflammatory and antioxidant effects, respectively.^22^ To date, however, there is no evidence that Cel protects against neonatal HI brain injury. In this study, we investigate the neuroprotective effects of Cel using in vivo and in vitro models of HI brain injury and investigate the potential mechanisms of action. Results indicate that Cel is a promising new therapeutic drug for treating HI brain injury in neonates.

## Materials and methods

### Neonatal HI brain injury model and drug administration

**Fig. 1:**
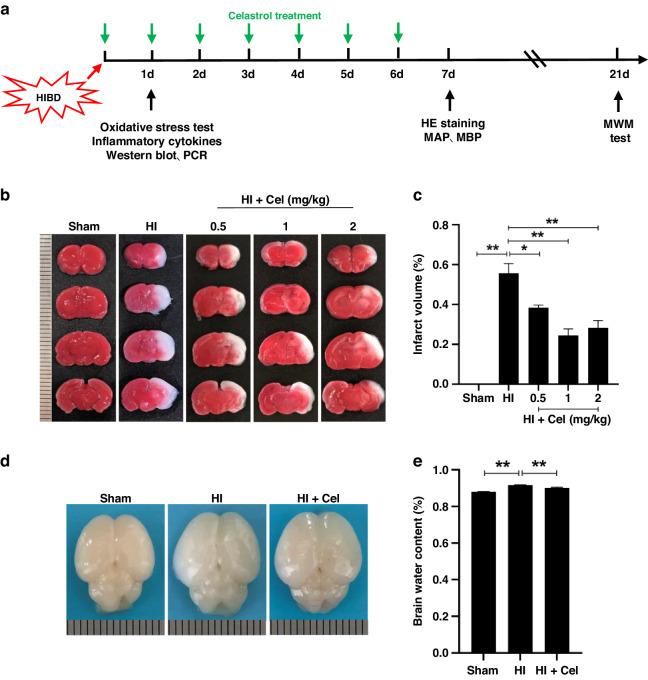
Cel treatment reduced brain damage in neonatal rats 1-day post HI. **a** Diagram of the experimental design. **b** Representative images of TTC-stained coronal brain section 1 day after HI damage. **c** Quantitative analysis of infarct volumes revealed by TTC staining. ^∗^*p* < 0.05 and ^∗∗^*p* < 0.01 vs. Sham or HI (*n* = 4). **d** Representative images of brains isolated from each group 24 h after HI damage. **e** The wet/dry ratio for each group. ^∗∗^*p* < 0.01 vs. Sham or HI (*n* = 5). Scale bar, 1 mm.

Sprague-Dawley (SD) rats (200–250 g) were purchased from the Animal Center of the Chinese Academy of Sciences (Shanghai, China), following guidelines for the Care and Use of Laboratory Animals of the National Institute of Health, and approved by the Laboratory Animal Ethics Committee of Wenzhou Medical University. Adult SD rats were allowed to mate freely, and postnatal day 7 (P7) male pups were used for in vivo experiments. The Rice-Vannucci model was established as described,^23^ with modifications. Briefly, P7 pups were completely anesthetized with isoflurane. The left common carotid artery was separated, ligated, and cut within 5 min. After the surgery, pups were allowed to recover in the dam for 2 h. Then, pups were placed into a 37.5 °C constant temperature environment with humid mixed gas (8% oxygen and 92% nitrogen with a flow rate of 3 L/min) for 2.5 h. Rats without ligation of the common carotid artery and hypoxia were in the Sham group. Celastrol (Cel, C107672, Aladdin Chemicals, Shanghai, China) was dissolved in DMSO and diluted with phosphate-buffered saline (PBS). The final concentration of DMSO in Cel solution was 1%. The Cel treatment group received different doses of Cel (0.5 mg/kg, 1 mg/kg, and 2 mg/kg) immediately after hypoxia through intraperitoneal injection every 24 h until the pups were euthanized to determine the most effective concentration. The same volume of solvent containing 1% DMSO was used to treat both Sham and HI groups to exclude the potential effect of 1% DMSO on the results (Fig. S1a, b). The AMPK inhibitor, Compound C (S7306, Selleck, Huston, TX), was dissolved in PBS (1 mg/mL) and 5 μl of the solution (5 ug Compound C per pup) was delivered via intracerebroventricular (icv) injection in a pup by using a stereotaxic apparatus (68045, RWD, China) 30 min before hypoxia. The diagram of the experimental design is shown in Fig. 1a.

### Infarct volume measurement and brain water content

Cerebral infarct volume was measured using 2,3,5-triphenyl tetrazolium chloride (TTC) (T8170, Solarbio Biotechnology) staining as described.^24^ In brief, 24 h after HI brain injury, pups in each group were anesthetized and perfused with normal saline. Tissues were collected and sectioned into 2 mm thick coronal slices. Samples were then immersed in 1% TTC solution at 37 °C in the dark for 30 min, followed by fixation with 4% paraformaldehyde for 24 h. ImageJ software (National Institutes of Health, Bethesda, MD) was used to calculate the cerebral infarct volume. Brain water content was measured to evaluate the degree of cerebral edema using the dry-wet ratio method as described.^25^ The left (i.e., injured) hemisphere was isolated and weighed to acquire the wet weight. The brain tissue was placed into an electrothermal oven at 70 °C for 48 h to obtain the dry weight.

### Detection of oxidative stress

Twenty-four hours after HI brain injury, rats were euthanized after deep anesthesia and the left cerebral cortex was removed and tissue homogenates (10%) were made with extract liquid. PC12 cells were collected and ultrasonically lysed 24 h after CoCl_2_ treatment. The supernatants were collected, and commercially available kits (Solarbio Biotechnology, Beijing, China) were used to detect the activity or level of various oxidative stress markers such as malondialdehyde (MDA), catalase (CAT), and glutathione (GSH).

### Histological staining

Seven days after the HI injury, rats were anesthetized with isoflurane and subjected to cardiac perfusion with 20 mL of normal saline and 20 mL of 4% PFA. After gradient dehydration, samples were embedded in paraffin and cut into 5 μm coronal sections for subsequent histological staining. Brain slices were stained with hematoxylin-eosin (HE) or Nissl staining (Solarbio Biotechnology). Bright-field images were obtained using a light microscope (Nikon, Japan) and images were analyzed using ImageJ software.

### Immunofluorescence staining

For in vivo experiments, brain sections harvested 24 h after HI brain damage were deparaffinized and antigen repaired after alcohol gradient hydration. Tissue sections were incubated in 10% BSA with 0.3% Triton X-100 for 1 h at room temperature. Sections were then incubated with primary antibodies (listed in Table S1) at 4 °C overnight, and then incubated with FITC-conjugated Goat Anti-Rabbit IgG secondary antibody (1:200, SA00003-2, Proteintech, Wuhan, China) at 37 °C for 1 h. Sections were analyzed and images were captured using a fluorescence microscope (Ni-U, Nikon, Japan).

### Morris water maze (MWM) test

The MWM test was conducted 21 days after the HI injury as described.^26^ In the MWM test, animals are forced to swim to find a platform that is hidden underwater. In brief, rats swim in a black circular pool (140 cm in diameter and 50 cm in height) that is placed in a room protected from light and noise. The pool is filled with water to a height of 1 cm above the top of the movable platform (diameter of 15 cm) and divided into four equal quadrants. During the first 5 days, each rat was trained once a day to swim in four quadrants for 60 seconds at a time to estimate their place navigation ability. After that, the rat was guided by the researcher to stay on the platform for 15 s. Then, the escape latency was recorded as the exact time that the rat found the hidden platform within 60 seconds. On Day 6, the platform was removed and all rats were allowed to swim for 60 seconds. During this time, the frequencies of finding the platform were recorded by DigBehv (Shanghai Jilang Software Technology Co., LTD).

### Cell culture and treatment

Differentiated PC12 cells were purchased from the Chinese Academy of Sciences (Shanghai, China). Cells were cultured in Dulbecco’s modified eagle medium (DMEM) supplemented with 10% FBS (Gibco) at 37 °C in a humidified incubator with a continuous 5% CO_2_ supply. In vitro cytotoxicity tests were performed after hypoxia induction using CoCl_2_ at the optimal concentration of 1 mM (based on the results of a cell viability assay). Cel was dissolved in DMSO to make a stock concentration of 10 mM. Based on initial results using different concentrations of Cel, 0.5 μM Cel was selected as the optimal concentration in subsequent experiments. PC12 cells were pretreated with Cel for 2 h before exposure to CoCl_2_. The Control group (not stimulated with CoCl_2_ and Cel) and the CoCl_2_ group (cultured without Cel) were incubated with a medium containing DMSO (0.05% v/v).

### Short-interfering RNA (siRNA) transfection

PC12 cells were transfected with 10 nM AMPKα-targeting siRNA (5′-GATGTCAGATGGTGAATTT-3′, RiboBio, Guangzhou, China) or control siRNA using opti-MEM (Gibco, Grand Island, NY) and Lipofectamine 3000 Transfection Reagent (Invitrogen, Carlsbad, CA).

### Annexin V and PI assay

Annexin V-PI apoptosis detection kit (C1062, Beyotime, Shanghai, China) was used for apoptosis analysis according to the protocol provided. Cells were plated in 24-well plates and washed with PBS once after drug treatment. Then, 195 μL of binding buffer was added to each well, and the cells were further stained with 5 μL of Annexin V-FITC and 10 μL of PI solution for 20 min in the dark at room temperature. Finally, the nuclei were stained with Hoechst and the cells were imaged using a fluorescence microscope (Ni-U; Nikon, Tokyo, Japan).

### Measurement of mitochondrial ROS

Mitochondrial ROS levels were analyzed using the MitoSOX Red mitochondrial superoxide indicator (Yeasen, Shanghai, China). In brief, MitoSOX Red was diluted using Hank’s balanced salt solution (HBSS) to a final concentration of 5 μM. Treated PC12 cells were washed with HBSS and incubated with diluted MitoSOX Red at 37 °C for 30 min. Extracellular MitoSOX Red was then completely removed by washing with HBSS. Cell nuclei were stained with DAPI in the dark. Finally, fluorescence was observed using a fluorescence microscope.

### RNA sequencing

Control or CoCl2-treated PC12 cells were sent to Beijing Genomics Institute (BGI, Shenzhen) for RNA sequencing (3 biological replicates for each treatment group). To gain insight into the critical pathways and biological processes involved in HI brain injury, Kyoto Encyclopedia of Gene and Genome (KEGG) and Gene ontology (GO) analysis of annotated different expression genes was performed by Phyper (https://en.wikipedia.org/wiki/Hypergeometric_distribution) based on Hypergeometric test. The significant levels of terms and pathways were confirmed at a threshold of *p* < 0.05.

### RNA extraction and quantitative real-time PCR (qRT-PCR)

Total RNA was extracted from samples with TRIzol reagent (Invitrogen, Carlsbad, CA). cDNA was obtained using a PrimeScript™ RT Master Mix kit (Takara Bio Inc., Japan). qRT-PCR was then performed on a CFX96 Optics Module (Bio-Rad, Singapore) using the TB Green Premix Ex Taq™ II kit (Takara Bio Inc., Japan). β-actin was used as an endogenous control. The 2^–ΔΔCt^ method was utilized to quantify target gene expression. Primers are listed in Table S2.

### Western blot analysis

Samples of brain tissue or PC12 cells were homogenized in RIPA lysis buffer supplemented with phenylmethane-sulfonyl fluoride and phosphatase inhibitors. Nuclear proteins were extracted from tissue homogenates using a nuclear extraction kit (R0050; Solarbio Biotechnology). For determining protein levels and levels of phosphorylation, equal quantities of proteins (60 μg in vivo and 30 μg in vitro) were separated using 10–12% SDS-PAGE gels and transferred to PVDF membranes. After blocking with 5% nonfat milk for 2 h at room temperature, protein blots were probed with primary antibodies, such as those against AMPKα, p-AMPKα, NLRP3, Nrf-2, TXNIP, heme oxygenase-1 (HO-1), MAP-2, MBP, Iba-1, TNF-α, IL-18, IL-6, IL-1β, β-actin and Lamin B1 (listed in Table S1). On the second day, membranes were incubated with HRP-Goat Anti-Rabbit IgG (1:5000, SA00001-2, Proteintech, Wuhan, China) for 2 h at room temperature. Protein bands were visualized using enhanced chemiluminescence (ECL) reagents (Bio-Rad, Hercules, CA). Lastly, densitometry values were analyzed using ImageJ software.

### Statistical analysis

GraphPad Prism 7.0 (GraphPad Software Inc., San Diego, CA) was used for statistical analyses. Quantitative data are presented as the mean ± SEM. The Student’s unpaired *t*-test was used to compare the two groups. Statistical significance was determined using the one-way analysis of variance (ANOVA) test followed by Tukey’s test when more than two groups were analyzed. *p* < 0.05 was considered statistically significant. *N* represents the number of replicates in each experiment.

## Results

### Cel ameliorates brain damage and histopathological changes, accelerates axonal repair, and promotes remyelination in neonatal rats subjected to HI

To investigate whether Cel could reduce brain damage resulting from HI, 0.5 mg/kg, 1 mg/kg, 2 mg/kg Cel was administered by intraperitoneal injection into HI-induced rats. Infarct volumes were then quantified in the Sham, HI, and HI + Cel groups. This analysis demonstrated that 1 mg/kg Cel is the most effective dose at reducing brain infarct volumes in HI-induced rats (Fig. 1b, c). Therefore, 1 mg/kg Cel was selected for subsequent experiments. We also imaged the brains of these neonatal rats 24 h after HI injury. There was more edema in the injured hemisphere of the HI group compared with the Sham group (Fig. 1d). Quantitative analysis of brain water content further showed that Cel alleviated brain liquefaction induced by HI injury (Fig. 1e). These data demonstrate that Cel treatment alleviated brain injury in neonatal rats subjected to HI.

**Fig. 2:**
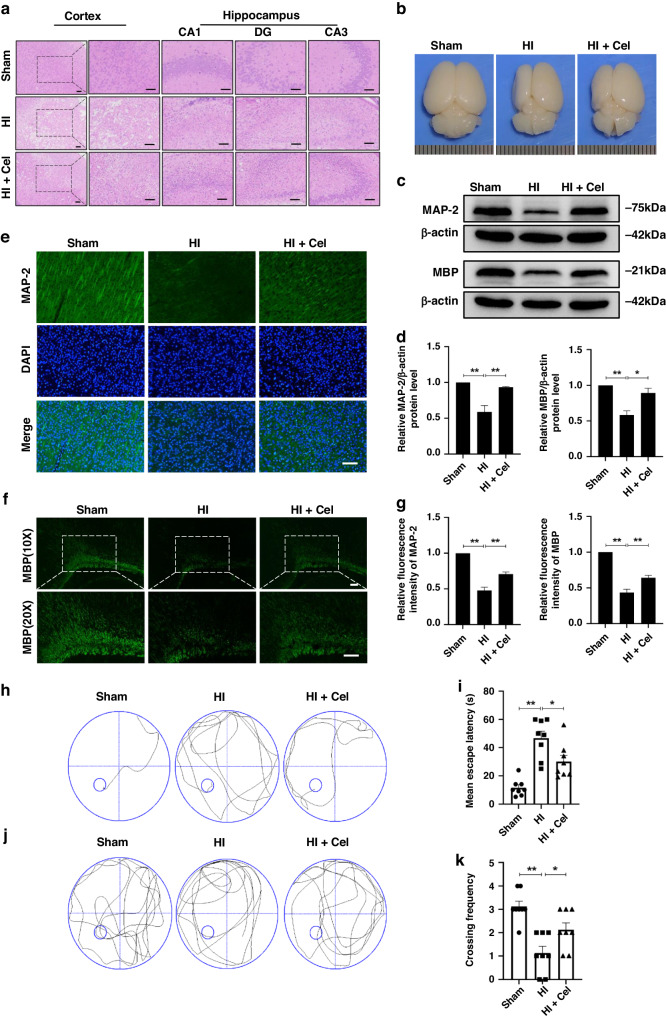
Cel ameliorated the histopathological changes, accelerated axonal repair and remyelination, and improved learning and memory in HI-injured rats. **a** Representative images of H&E stained cortex, as well as the CA1 region, CA3 region, and DG of the hippocampus 7 days after HI injury. Scale bar, 100 μm. **b** Images of brains isolated from each group 7 days post HI treatment. **c** Levels of MAP-2 and MBP protein were evaluated by western blot analysis of brain tissues 7 days post HI treatment. **d** Quantitative analysis of protein levels. ^∗^*p* < 0.05 and ^∗∗^*p* < 0.01 vs. Sham or HI (*n* = 3). **e** Representative immunofluorescence images of MAP-2 (green) and DAPI (blue). **f** Representative immunofluorescence images of MBP (green) and DAPI (blue). Scale bar, 100 μm. **g** Quantitation of immunofluorescence staining for MAP-2 and MBP. **h** Representative swim route traces of rats from different groups as they attempted to locate a hidden platform (21 days after HI injury). **i** Quantitative analysis of mean escape latency in the MWM tests for different groups 21 days after HI injury. ^∗^*p* < 0.05 and ^∗∗^*p* < 0^.^01 vs. Sham or HI (*n* = 8)^.^ **j** Representative swim route traces of rats from different groups after removal of the platform. **k** Quantitative analysis of the frequency at which rats crossed the original location of the platform in 60 s trails. ^∗^*p* < 0.05 and ^∗∗^*p* < 0.01 vs. Sham or HI (*n* = 8).

Then, we performed HE staining 7 days after HI injury to assess neuronal morphology. The extent of HI injury was assessed by observing the numbers and morphology of cells in different brain regions. In the Sham group, neurons in the cortex, as well as the CA1 region, CA3 region, and dentate gyrus (DG) of the hippocampus were regularly arranged. Nuclei were clear and intact with a round or oval shape. In the HI group, neurons were disordered with pyknotic nuclei, and a notable absence of neurons was observed. Cel treatment decreased the extent of neuronal degeneration and necrosis and increased the number of neurons (Fig. 2a). We also assessed overall brain anatomical structure 7 days after HI damage. A great deal of atrophy was apparent in the injured hemisphere. This atrophy was relieved by Cel intervention compared with the HI group (Fig. 2b).

MAP-2 is a primary regulator of the cytoskeleton within neuronal dendrites, serving as a robust somatodendritic marker.^27^ MBP is a key component of the myelin sheath in the central nervous system and serves as a marker of oligodendrocytes.^28^ Western blotting (Fig. 2c) and immunohistochemical staining for MAP-2 (Fig. 2e) and MBP (Fig. 2f) were performed to investigate whether Cel accelerated axonal repair and remyelination 7 days post-HI injury. These analyses revealed that the levels of MAP-2 and MBP were lower in the HI group compared with the Sham group. Cel reversed this trend (Fig. 2d, g). These results indicate that Cel reduced neuronal loss, promoted morphological recovery, stabilized microtubule function, and promoted myelination after HI brain injury.

### Cel treatment improves learning and memory in HI-injured rats

To assess the effects of Cel on learning and memory following HI-induced brain injury, rats were subjected to the Morris water maze test 21 days after the HI insult. After 5 days of training, rats were subjected to the spatial acquisition test (Fig. 2h). Compared with the Sham group, rats in the HI group exhibited a longer mean escape latency (46.75 ± 4.87 s vs. 11.50 ± 2.12 s, *p* < 0.01). Cel treatment reversed this effect (30.13 ± 4.41 s) in HI-induced rats (Fig. 2i). We then removed the platform and assessed spatial memory by recording the frequency with which rats crossed over the original position of the platform (Fig. 2j). The HI group exhibited impaired memory compared with the Sham group. The Cel treatment group crossed the platform location more frequently (Fig. 2k). These data suggest that Cel administration improved learning and cognitive function in rats following HI insult.

### Cel reduces CoCl_2_-induced cell injury in PC12 cells

**Fig. 3:**
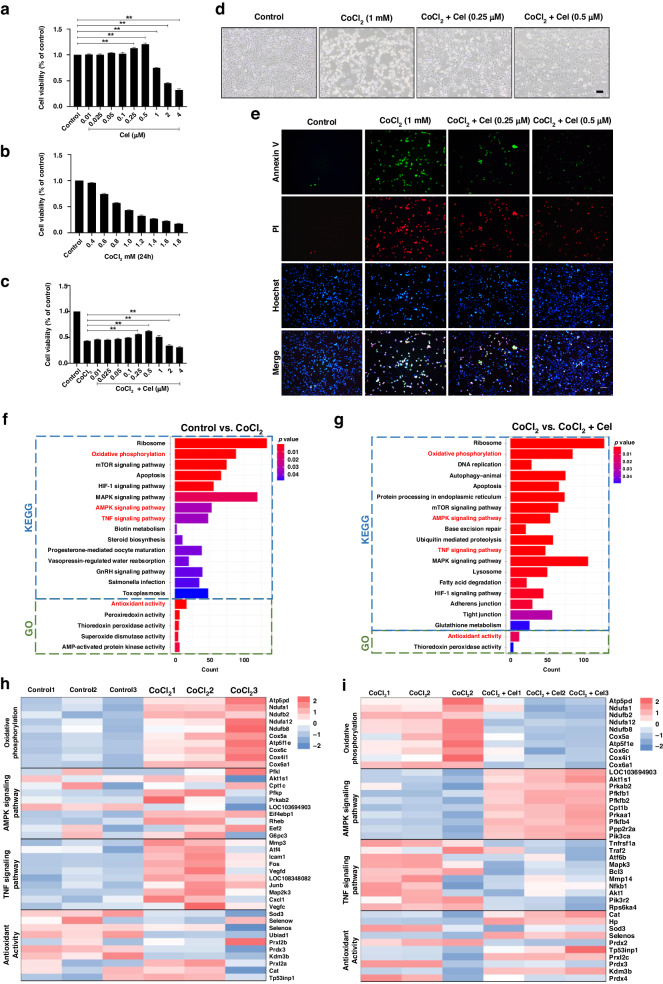
Cel reduced CoCl_2_-induced cell injury in PC12 cells and screening for candidate pathways. **a** The pharmacologically safe dose at which Cel maintains cell viability was assessed using the CCK-8 assay. **b** PC12 cells were treated with different doses of CoCl_2_ for 24 h and then subjected to the CCK-8 assay. **c** Dose-dependent effects of Cel on cell viability following 24 h of CoCl_2_ treatment. **d** Effects of different concentrations of Cel on cell morphology following 24 h of CoCl_2_ treatment. Scale bar, 100 μm. **e** Apoptosis in CoCl_2_-injured PC12 with or without Cel treatment as measured by Annexin V-FITC (green) and PI (red) detection. Scale bar, 100 μm. **f** The 20 most enriched pathways were analyzed using KEGG (blue) and GO (green) databases (*p* < 0.05) in comparisons of the Control vs. CoCl_2_ groups. **g** The 20 most enriched pathways were analyzed using KEGG (blue) and GO (green) databases (*p* < 0.05) in comparisons of the CoCl_2_ vs. CoCl_2_ + Cel groups. **h** Heatmap showing each top 10 DEGs of signaling pathways in the comparison of the Control and CoCl_2_ group. **i** Heatmap showing each top 10 DEGs of signaling pathways in the comparison of the CoCl_2_ and CoCl_2_ + Cel group.

To explore the in vivo effects described above, PC12 was exposed to CoCl_2_ to simulate HI in vitro. We first assessed the cytotoxicity of Cel on PC12 cells using the CCK-8 assay. Cel (0–0.5 μM) had no obvious adverse effects on cell viability (Fig. 3a). We then assessed the cytotoxicity of CoCl_2_ on PC12 cells. Treatment with different concentrations of CoCl_2_ (0–1.8 mM) for 24 h resulted in a dose-dependent decrease in cell viability compared with controls. In addition, the viability of PC12 cells exposed to 1.0 mM CoCl_2_ for 24 h decreased to 43.44 ± 1.03% (Fig. 3b). Based on this result, we chose 1.0 mM CoCl_2_ as the concentration used for subsequent experiments. Finally, we pretreated PC12 cells with different concentrations of Cel (0–4 μM) for 2 h before subjecting them to 1.0 mM of CoCl_2_ for 24 h. Cell viability was optimal at a dose of 0.5 μM Cel (Fig. 3c). Images of PC12 cells were captured immediately after 24 h of CoCl_2_ treatment. Treating PC12 cells with CoCl_2_ resulted in cell shrinkage and rounding. Cel treatment reduced this effect, with the most dramatic effects seen at 0.5 μM (Fig. 3d). Meanwhile, CoCl_2_ treatment notably increased the early apoptotic (Annexin V-positive and PI-negative staining) cells and late apoptotic (Annexin V-positive and PI-positive staining) cells, which was reversed by Cel intervention (Fig. 3e).

### Cel down-regulates acute inflammatory responses and oxidative stress via AMPKα/Nrf2/TXNIP signaling pathway

To explore the mechanism underlying the neuroprotective effects of Cel, cultured PC12 cells were exposed to the hypoxia mimetic CoCl_2_ in the presence or absence of Cel and RNA sequencing analysis was performed. We identified differentially expressed genes (DEGs) and conducted GO and KEGG analyses. GO and KEGG enrichment analyses revealed statistical significance of 109 KEGG and 10 GO between the Control and CoCl_2_ group, while 138 KEGG and 9 GO between the CoCl_2_ and CoCl_2_+Cel group. Importantly, 4 of the top 20 signaling pathways including oxidative phosphorylation, AMPK signaling pathway, TNF signaling pathway and antioxidant activity were selected to be the mechanism related to the potential effects of Cel (Fig. 3f, g). We listed each top 10 DEGs, and identified that CoCl_2_ led to the upregulation of genes associated with the oxidative phosphorylation, AMPK signaling pathway, and TNF signaling pathway while inhibiting the pathway associated with the antioxidant activity (Fig. 3h). Cel administration reversed these trends while further upregulated the AMPK signaling pathway (Fig. 3i). We therefore surmised that Cel might inhibit oxidative stress and inflammation by activating AMPK signaling.

**Fig. 4:**
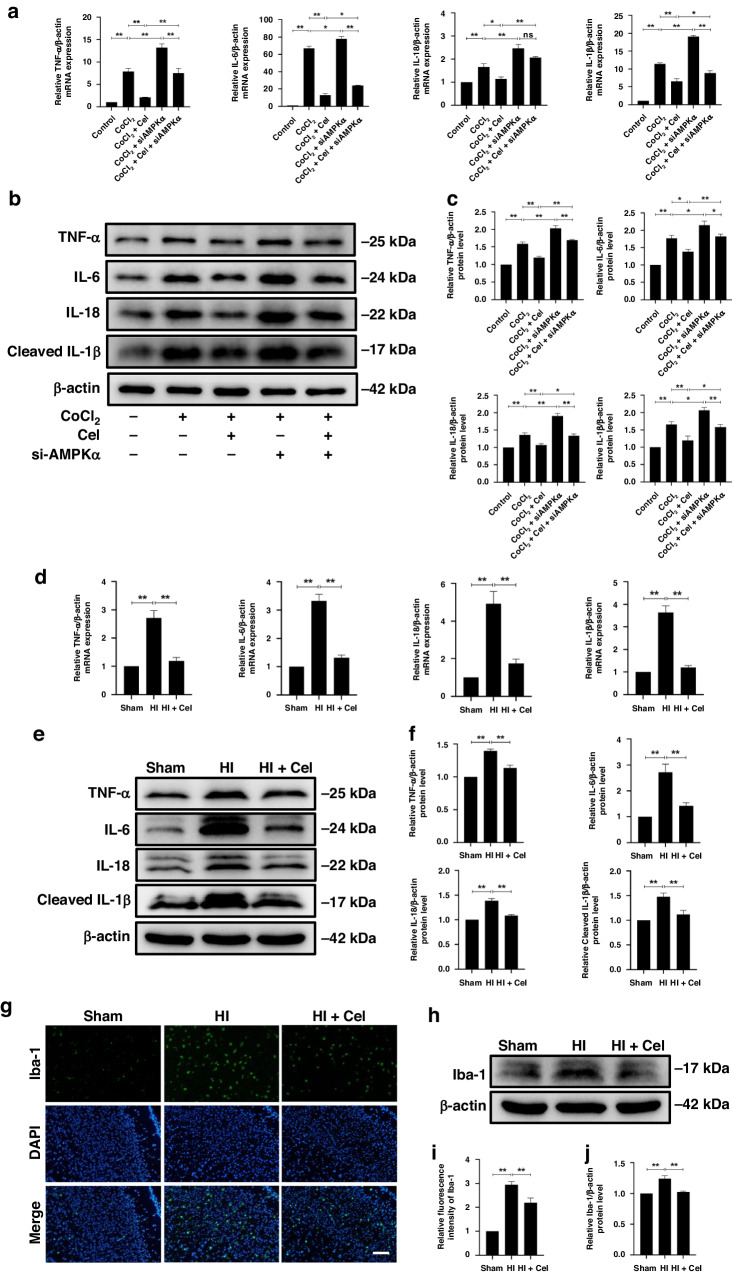
Cel down-regulates acute inflammatory responses. **a** MRNA expression levels of TNF-α, IL-6, IL-18, and IL-1β by qRT-PCR analysis in PC12 cells. ^∗^*p* < 0.05 and ^**^p < 0.01 indicate significant differences (*n* = 5). **b** Expression levels of TNF-α, IL-6, IL-18, and IL-1β proteins were evaluated by western blot in PC12 cells. **c** Quantitative analysis of protein levels. ^∗^*p* < 0.05 and ^**^p < 0^.^01 indicate significant differences (*n* = 5). **d** MRNA expression levels of TNF-α, IL-6, IL-18, and IL-1β by qRT-PCR analysis in brain tissues 24 h after HI brain injury. ^∗∗^*p* < 0.01 vs. Sham or HI (*n* = 5). **e** Protein levels of TNF-α, IL-6, IL-18, and cleaved IL-1β were measured by western blot 24 h after HI injury. **f** Quantitative analysis of protein levels. ^∗∗^*p* < 0.01 vs. Sham or HI (*n* = 5). **g** Representative immunofluorescence images of Iba-1 (green) and DAPI (blue) 24 h after HI injury. Scale bar, 100 μm. **h** Levels of Iba-1 protein were measured by western blot 24 h after HI injury. **i** Quantitation of immunofluorescence staining for Iba-1. **j** Quantitative analysis of Iba-1 protein level. ^∗∗^*p* < 0.01 vs. Sham or HI (*n* = 5).

Indeed, we found that Cel treatment substantially suppressed the mRNA levels and protein expressions of TNF-α, IL-6, IL-18, IL-1β in CoCl_2_-treated PC12 cells (Fig. 4a-c). Consistent with these data, the treatment of HI-induced rats with Cel robustly downregulated inflammatory biomarkers, such as TNF-α, IL-6, IL-18, and IL-1β both by qRT-PCR (Fig. 4d) and western blot analysis (Fig. 4e, f). Besides, the inflammatory response in the injured hemisphere of the brain by HI damage is characterized by abundant inflammatory cytokines release largely due to the activation of microglia.^29^ Therefore, immunofluorescence staining was conducted to detect the expression of Iba-1, which is a classical bio-marker of microglia activation (Fig. 4g).^30^ Results showed that HI increased the number of Iba-1-positive cells in the injured hemisphere and that Cel treatment alleviated this effect (Fig. 4i). Moreover, western blot analysis (Fig. 4h) showed that levels of Iba-1 in the injured hemisphere were upregulated in the HI group compared with the Sham group. Cel administration reversed this effect in HI-induced rats (Fig. 4j).

**Fig. 5:**
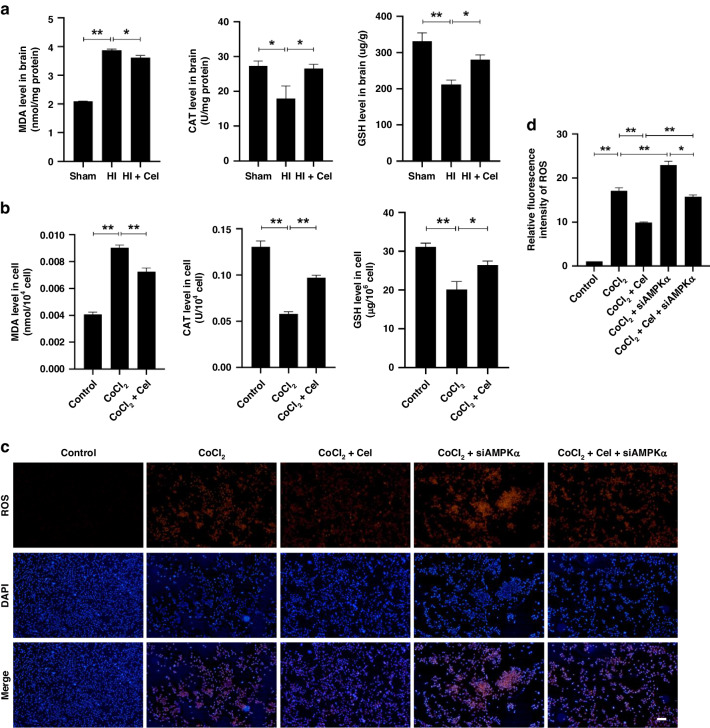
Cel treatment downregulated levels of oxidative stress. **a** Levels of MDA, CAT, and GSH in brain tissues 24 h after HI brain injury. ^∗^*p* < 0.05 and ^∗∗^*p* < 0.01 vs. Sham or HI (*n* = 5). **b** Levels of MDA, CAT, and GSH in PC12 cells 24 h after CoCl_2_ treatment. ^∗^*p* < 0.05 and ^∗∗^*p* < 0^.^01 vs. Control or CoCl_2_ (*n* = 5). **c** Levels of mitochondrial ROS were determined for each group using MitoSox (red) and DAPI (blue) staining in PC12 cells. Mean ± SEM. ^*^p < 0.05, ^**^p < 0.01 indicate significant differences. Scale bar 100 μm. **d** Quantitation of immunofluorescence staining for ROS in PC12 cells.

To investigate whether Cel reduced oxidative stress, we then assessed the levels of MDA, CAT, and GSH. In the rat model, we found that HI aggravated oxidative stress as revealed by the increased concentration of MDA and decreased activities of CAT and GSH in the injured hemisphere. These changes were reversed by Cel intervention (Fig. 5a). In parallel, the in vitro results also showed that MDA level was dramatically increased and accompanied by the decline of CAT and GSH after CoCl_2_ treatment, whereas Cel treatment reversed all these effects (Fig. 5b). We also measured mitochondrial ROS production using the MitoSOX fluorescent probe assay in PC12 cells (Fig. 5c). Cel pretreatment attenuates the increased ROS levels induced by CoCl_2_ (Fig. 5d), suggesting that Cel can attenuate oxidative stress.

**Fig. 6:**
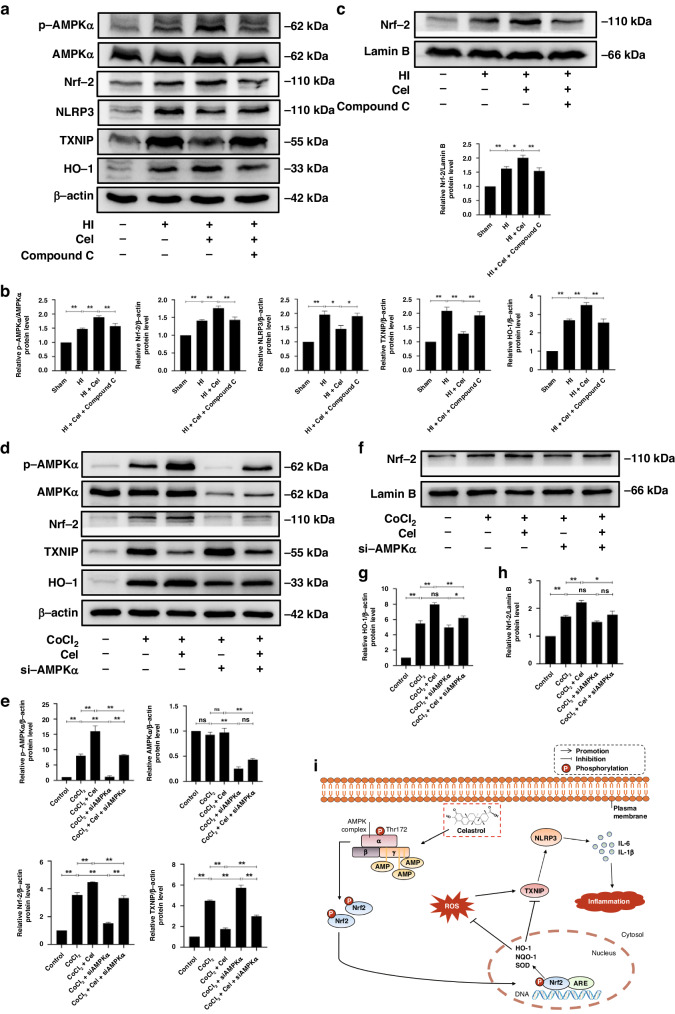
Cel treatment regulated the AMPKα/Nrf2/TXNIP pathway in neonatal rats following HI brain injury. **a** Protein levels of p-AMPKα, AMPKα, Nrf-2, NLRP3, TXNIP, and HO-1 were evaluated by western blot. **b** Quantitative analysis of protein levels. ^∗^*p* < 0.05 and ^∗∗^*p* < 0.01 vs. Sham or HI (*n* = 5). **c** Western blot analysis and quantification of nuclear Nrf2 in rats. ^∗^*p* < 0.05 and ^∗∗^*p* < 0^.^01 vs. Sham or HI (*n* = 5)^.^ **d** Expression levels of p-AMPKα, AMPKα, Nrf**-**2, TXNIP, and HO-1 protein were evaluated by western blot. **e**, **g** Quantification of western blot data. ^**^p < 0.01 indicates significant differences^.^ **f**, **h** Western blot analysis and quantification of nuclear Nrf2 in PC12 cells. ^*^p < 0.05, ^**^p < 0.01 indicate significant differences. **i** Mechanistic diagram of Cel-mediated neuroprotective effects following HI brain injury.

Based on the above results, we investigated the effect of Cel on AMPKα signaling. Compared with the Sham group, levels of p-AMPKα/AMPKα were upregulated by HI. Cel administration further upregulated levels of p-AMPKα/AMPKα (Fig. 6a). We then explored the downstream antioxidant and anti-inflammatory signals that were controlled by the Cel-AMPKα signaling and found upregulated levels of Nrf2 and HO-1 but decreased NLRP3 and TXNIP levels in rats (Fig. 6a, b). Moreover, assessing nuclear proteins via western blot indicated that HI injury promoted the nuclear transfer of Nrf2. Levels of nuclear Nrf2 were higher after Cel treatment (Fig. 6c). These results were further validated in PC12-based experiments in which Cel pretreatment downregulated levels of TXNIP protein, but upregulated p-AMPKα, Nrf2, and HO-1 (Fig. 6d–h).

To validate the role of AMPKα in Cel-mediated alleviation of inflammatory and oxidative stress induced by HI, we used an AMPK selective inhibitor (Compound C)^31^ in rats. We found that the inhibition of AMPK directly downregulated Nrf2 and HO-1 expressions while upregulated NLRP3 and TXNIP expressions (Fig. S2a, b). Moreover, we found that Compound C largely compromised the increases in p-AMPKα, Nrf2, and HO-1 and the decreases in NLRP3 and TXNIP by Cel treatment in the rat model (Fig. 6a). Consistent with these data, the inhibition of inflammatory biomarkers, such as TNF-α, IL-6, IL-18, and IL-1β after Cel treatment in PC12 cells was reversed by the administration of AMPKα siRNA (Fig. 4a, b). Furthermore, in the MitoSOX fluorescent probe experiment in PC12 cells, Cel treatment significantly inhibits the production of ROS, whereas silencing of AMPKα mitigated this effect (Fig. 5c). Besides, silencing AMPKα counteracted the increase in p-AMPKα, Nrf2, and HO-1, as well as the decrease in TXNIP, resulting from Cel treatment of CoCl_2_-treated PC12 cells (Fig. 6d).

Taken together, these analyses provide compelling evidence that Cel decreases inflammation and oxidative stress through the AMPKα/Nrf2/TXNIP pathway (as shown in Fig. 6i), thereby alleviating neonatal HI injury. Cel therefore represents a promising therapeutic agent for treating HIE.

## Discussion

HIE is one of the leading causes of neonatal morbidity and mortality worldwide, even with the application of hypothermia.^32^ HIE is not a single event but is an ongoing process that leads to the death of neurons within hours to days of the initial injury.^33^ Current efforts are focusing on finding effective drugs for treating HIE.

Cel is a pentacyclic triterpene extracted from *Tripterygium Wilfordi* with a proven structure widely used in China and other Asian countries to treat a variety of chronic inflammatory diseases.^34^ Recently, numerous studies have shown that Cel has antioxidant, anti-inflammatory, and anti-tumor activities.^35–37^ Jiang et al. found that both the anti-inflammatory and anti-apoptotic functions of Cel are related to IL-33/ST2-mediated M2 polarization.^18^ Cel also protects against neurodegenerative diseases by alleviating oxidative stress and inflammation to maintain the integrity of the blood–brain barrier.^16,38^ To date, however, the effect and potential mechanism of Cel in the context of neonatal HI brain injury have not been explored. Here, we investigated the neuroprotective effects of Cel in HI-induced brain damage and explored the underlying mechanism using both in vitro and in vivo models (CoCl_2_-treated PC12 cells and rats subjected to neonatal HI brain injury, respectively). Our results revealed that Cel administration reduced the extent of cerebral infarction and alleviated brain edema following HI injury. Further experiments revealed that Cel ameliorated histopathological changes, accelerated axonal repair, and promoted remyelination 7 days after injury. Cel treatment also improved learning and memory in rats subjected to HI. For PC12 cells treated with CoCl_2_, Cel promoted cell survival. It is worth noting that Cel at concentrations >0.5 μM inhibited the proliferation of PC12 cells. This may relate to the antiproliferative effect of Cel in a wide range of human cancer cells, including glioma, breast cancer, and head and neck carcinoma.^39^ Boridy et al. found that Cel can target the proteotoxic stress responses by inhibiting the expression of heat-shock protein 90 (HSP90) and promote cell death through the ROS/c-Jun N-terminal kinase (JNK) pathway in human glioblastoma cells.^40^

We used RNA sequencing analysis to elucidate the mechanisms of HI-induced brain injury and to gain insights into the potential signaling pathways regulated by Cel in this context. Bioinformatic analysis of RNA sequencing data from PC12 cells subjected to different treatments revealed that Cel activated pathways related to AMPK signaling and antioxidant activity, whereas Cel inhibited TNF signaling and oxidative phosphorylation. These findings reveal that Cel may exert pleiotropic effects against HI brain injury. Moreover, our findings revealed that AMPKα, Nrf2, and TXNIP may underlie the pleiotropic functions of Cel in HI brain injury. Specifically, Cel may protect against HIE by regulating the AMPKα/Nrf2/TXNIP pathway.

AMPK is a heterotrimeric complex consisting of a catalytic α­subunit and two regulatory subunits (β and γ) and is responsible for maintaining the balance between the production and consumption of adenosine-triphosphate (ATP) in cells.^41^ Several factors such as hypoglycemia, hypoxia, ischemia, and heat shock, which each can result in an insufficient supply of cellular energy, activate AMPK via the α subunit, and modulate many biological processes, including inflammation and oxidative stress.^42,43^ AMPK activation can ameliorate oxidative stress and inflammation in many central nervous system diseases, and AMPK inhibition can aggravate brain damage.^44–46^ There is growing evidence that Cel is a strong inducer of AMPK.^47,48^ Cel activates AMPK in the skeletal muscle of rats and increases AMPK phosphorylation in hepatic stellate cells.^49,50^ In this study, we explored the protective effects of AMPKα in the context of HI injury and the underlying mechanisms. We showed that the ratio of p-AMPKα/AMPKα increased in the brain tissue of rats subjected to HI and in PC12 cells treated with CoCl_2_. In addition, Cel enhanced the phosphorylation of AMPKα, increased levels of Nrf2 and HO-1, and decreased levels of TXNIP and NLRP3, both in HI-induced rats and in CoCl_2_-treated PC12 cells. To confirm whether the protective effects of Cel against HI-induced brain injury are through the AMPK pathway, we administered AMPKα siRNA to PC12 cells treated with both CoCl_2_ and Cel. Interestingly, the neuroprotective effects of Cel were abrogated upon AMPKα knockdown. This was associated with reductions in Nrf2 and HO-1 levels and increases in TXNIP and NLRP3. In addition, the down-regulation of AMPKα aggravated CoCl_2_-induced inflammation and oxidative stress, which could be reversed by Cel treatment.

We have previously demonstrated important roles for oxidative stress and inflammation in neonatal HI brain injury.^25,51^ Multiple lines of evidence indicate that levels of Nrf2 protein are upregulated by AMPK activation and that translocation of Nrf2 to the nucleus enhances the antioxidant system to reduce oxidative stress in the brain. This occurs through activating downstream antioxidant enzymes, such as HO-1, superoxide dismutase (SOD), and NADPH quinone oxidoreductase 1 (NQO1).^52^ Recently, Hu et al. reported that Nrf2-knockout exacerbates HIE phenotypes in mice.^53^ Further analysis showed that Nrf2 activation attenuates inflammation and oxidative stress in HI-induced brain injury.^54^ Our findings here are consistent with these reports and reveal that Cel treatment increased levels of Nrf2 protein and a downstream target HO-1 in neonatal HI rats. Our findings further showed that Cel administration reduced levels of MDA, which is a gold standard marker for evaluating oxidative stress, and dramatically increased markers of antioxidant defense, such as CAT and GSH in brain tissues of HI rats. Earlier, He et al. showed that the antioxidant transcription factor Nrf2 represses TXNIP expression in response to oxidative stress.^55^ After oxidation of thioredoxin (TRX) by ROS, TXNIP dissociates from TRX to associate with NLRP3, activating inflammasomes to mediate inflammatory signaling.^56^ Therefore, TXNIP is thought to link oxidative stress and inflammation. Chen et al. showed that the upregulation of TXNIP activates the NLRP3 inflammasome and worsens HI brain injury in neonatal rats.^57^ Throughout this study, we demonstrated that Cel reduced HI-induced activation of TXNIP and NLRP3, as well as levels of inflammatory mediators including TNF-α, IL-6, IL-18, and IL-1β in neonatal rats. Similar anti-inflammatory effects of Cel were observed in PC12 cells treated with CoCl_2_, which is consistent with research into the effects of Cel on pancreatic β cells under high-glucose conditions.^58^

However, the present study has some limitations that need to be addressed in further studies. (i) Although our study demonstrated that AMPKα knocked upregulated inflammatory cytokines and oxidative stress, we did not investigate whether its activation had anti-inflammatory and antioxidant effects. (ii) Further in vitro experiments using primary neurons subjected to oxygen-glucose deprivation (OGD) would strengthen our present results.

## Supplementary information

Supplementary Materials

## Data Availability

RNA-Seq data was available in BioProject # PRJNA897944. Data supporting the present study are available from the corresponding author upon reasonable request.
