# Supplementary material for: Celastrol ameliorates hypoxic-ischemic brain injury in neonatal rats by reducing oxidative stress and inflammation
Source: Pediatr Res. 2024 May 20;96(7):1681–92. doi: 10.1038/s41390-024-03246-9 (PMC11772252; doi:10.1038/s41390-024-03246-9)
Supplement: Supplementary file 1 — Supplementary Materials [file 41390_2024_3246_MOESM1_ESM.pdf]

Supplementary Materials

Celastrol ameliorates hypoxic-ischemic brain injury in neonatal rats by reducing oxidative stress and inflammation

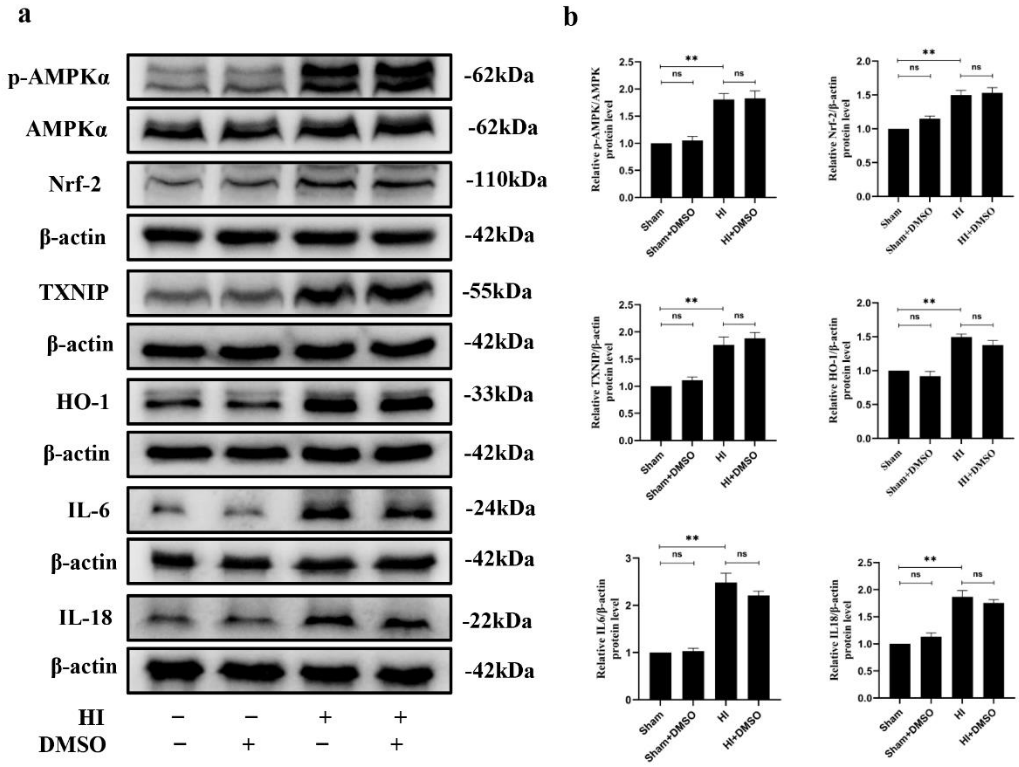

Fig. s1 Effects of 1% DMSO in neonatal rats post HI.

a Protein levels of p-AMPKα, AMPKα, Nrf-2, TXNIP, HO-1, IL-6 and IL18 were evaluated by western blot in neonatal rats after HI damage with or without 1% DMSO in solvent. **b** Quantitative analysis of protein levels. $^{**}p < 0.01$ vs. Sham (n = 4).

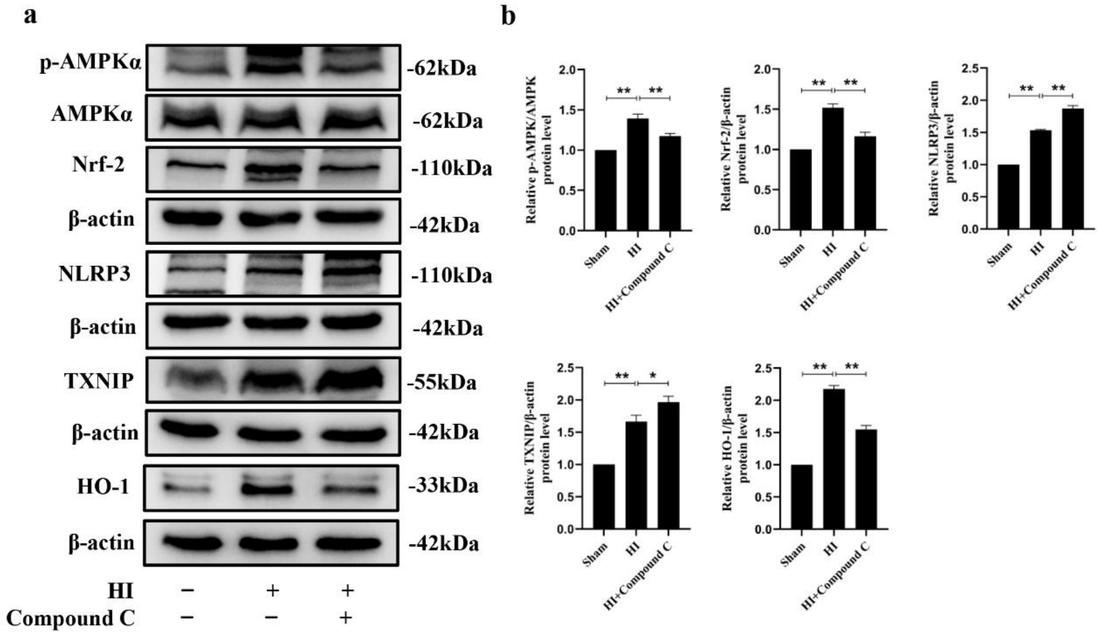

Fig. s2 AMPK inhibition suppressed anti-oxidative ability and upregulated inflammations in neonatal rats after HI.

a Protein levels of p-AMPKα, AMPKα, Nrf-2, NLRP3, TXNIP, and HO-1 were evaluated by western blot in neonatal rats 24 h after HI brain injury with or without the treatment of compound C. **b** Quantitative analysis of protein levels. $^{*}p < 0.05$ and $^{**}p < 0.01$ vs. Sham or HI (n = 5).

Table s1. Antibodies

Antibody	Vendor (City, State, catalogue)	Dilution	
		WB	IF
AMPKα	Cell Signaling Technology (Danvers, MA, 5831)	1:1000	ND
p-AMPKα	Cell Signaling Technology (Danvers, MA, 2535)	1:1000	ND
NLRP3	Abclonal (Wuhan, China, A5652)	1:1000	ND
Nrf-2	Proteintech (Wuhan, China, 16396-1-AP)	1:1000	1:200
TXNIP	Abcam (Cambridge, UK, ab188865)	1:1000	ND
HO-1	Proteintech (Wuhan, China, 10701-1-AP)	1:1000	ND
MAP-2	Abcam (Cambridge, UK, ab254264)	1:1000	1:2000
MBP	Abcam (Cambridge, UK, ab218011)	1:1000	1:5000
Iba-1	Abcam (Cambridge, UK, ab178846)	1:1000	1:1000
TNF-α	Affinity Biosciences (OH, USA, AF7014)	1:1000	ND
IL-18	Proteintech (Wuhan, China, 10663-1-AP)	1:1000	ND
IL-6	Affinity Biosciences (OH, USA, DF6087)	1:1000	ND
IL-1β	Affinity Biosciences (OH, USA, AF5103)	1:1000	ND
ACTB	Abclonal (Wuhan, China, AC026)	1:10000	ND
Lamin B1	Proteintech (Wuhan, China, 12987-1-AP)	1:10000	ND

ND = Not detected; WB = Western blot; IF: Immunofluorescence.

Table s2. Primer information

Primer Symbol	Gene name	Primer direction	Sequences (5'to 3')
TNF-α	Tumor necrosis factor alpha	Forward	GAGGTCAACCTGCCCAAGTA
		Reverse	GCTGGGTAGAGAACGGATGA
IL-6	Interleukin 6	Forward	CCAGTTGCCMDATTGGGACT
		Reverse	TCTGACAGTGCATCATCGCT
IL-18	Interleukin 18	Forward	CGAACAGCCAACGAATCCCAGAC
		Reverse	TCACAGATAGGGTCACAGCCAGTC
IL-1β	Interleukin 1 beta	Forward	AATCTCACAGCAGCATCTCGACAAG
		Reverse	TCCACGGGCAAGACATAGGTAGC
β-actin	Actin, beta (Actb)	Forward	AAGTCCCTCACCCTCCCAAAAG
		Reverse	AAGCAATGCTGTCACCTTCCC
